# Sleep Monitoring Using WatchPAT Device to Predict Recurrence of Major Depression in Patients at High Risk for Major Depression Disorder Recurrence: A Case Report

**DOI:** 10.3389/fpsyt.2021.572660

**Published:** 2021-06-25

**Authors:** Daniel Harlev, Ramit Ravona-Springer, Yonatan Nuriel, Eyal Fruchter

**Affiliations:** ^1^Rambam Health Care Campus, Haifa, Israel; ^2^Sheba Medical Center, Ramat Gan, Israel

**Keywords:** sleep, major depressive disorder, WatchPAT, rapid eye movement sleep, ambulatory

## Abstract

**Background:** Major depressive disorders are strongly correlated with alterations in sleep pattern and architecture, including changes in the Rapid Eye Movement (REM) phase. However, it is still unknown whether sleep alterations precede other depression-related symptoms, particularly in patients with recurrent depressive episodes at relapse risk.

**Case Presentation:** We initiated a study aimed at examining the value of ambulatory sleep monitoring using a WatchPAT device, in predicting recurrence of Major depression. Depression was assessed monthly with the Beck Depression Inventory version II (BDI-II). Here we present the case of a 63 years old woman, with a history of recurrent depressive episodes. AT the time of recruitment, she was asymptomatic, she experienced recurrence of Major depression 3 months into the study. We observed a significant reduction of the Rem Latency parameters 5 weeks prior to BDI-II score increase, reflecting major depressive episode recurrence.

**Conclusion:** Though our results are preliminary, they suggest that ambulatory sleep monitoring can be used as a simple and accessible tool, predicting recurrence of Major Depressive episodes in patients at high risk, thus enabling early treatment intervention.

## Background

Major depression is a serious disorder that leads to considerable disability and suffering worldwide. The lifetime prevalence of major depressive disorder (MDD) in the United States is 16.6% (1). MDD has severe consequences and is associated with increased rates of disability, morbidity and mortality (2). Approximately 25% of patients experience a recurrence of major depressive disorder within the first 6 months after being released from a hospital, 30 to 50% in the following 2 years, and 50 to 75% in 5 years. Generally, as patients experience increased number of depressive episodes, the time interval between them decreases, and the severity of each episode increases (3). Early intervention was associated with significant decrease of the overall length of the second depressive episode by ~4–5 months (4). Furthermore, early identification and treatment may prevent the development of a full depressive episode.

The literature considers Sleep disorders as an important component of major depression, inherent to the pathophysiology of the disorder. Disturbed sleep reported by as many as 90% of depressed subjects (5). Polysomnography (PSG) studies demonstrate specific depression-related changes in sleep architecture. Changes include a decrease in rapid eye movement (REM) sleep stage latency (time interval between sleep onset and the occurrence of the first REM sleep period) among others. The mentioned above REM sleep alterations might have prodromal and residual properties with respect to depressive episodes (6–10). In general, sleep pattern changes among patients with depression consist of impaired sleep onset and maintenance, early morning awakening as well as reduced deep sleep and disinhibition of REM sleep. Patient's complaints of insomnia consistent over a period of 2 weeks could be a useful marker of subsequent onset of major depression (11). However, subjective reporting is inherently variable and not always applicable, and a significant proportion of sleep disorder complaints are common in the general population. Therefore, the question arises about the predictive value of objectively measured sleep disorders.

Studies investigating relatives of patients with depression demonstrated that specific REM density changes are already present before the onset of the disorder and may predict its development (12–15). Recurrent depressed subjects present REM sleep disturbances before the onset of the treatment and soon after symptomatic remission independently of treatment method (16). However, this study did not examine whether patients can be monitored in the natural (ambulatory) environment, nor did it examine the observed changes over a sufficient period after remission.

A recent meta-analysis of the PSG sleep research literature on depression (9) demonstrated that REM latency and REM density discriminated depressed from non-depressed subjects convincingly with statistical significance; with respect to within subject comparisons looking at changes from the depressed to the remitted state only REM density remained unchanged. It was found that changes of REM sleep occur between 50 and 70% of subjects afflicted with major depression (5). When comparing patients suffering from MDD to those in remission, effect sizes were strongest for REM latency.

Thus, changes in sleep architecture, particularly REM latency, can potentially be used to predict recurrence of MDD, and consequently early introduction of treatment. However, sleep monitoring based on PSG has limited value in real life settings, and so clinical implications are limited.

Regarding monitoring sleep variables in ambulatory settings, it was found that new actigraphy algorithm of the WatchPAT device provides a reasonably accurate estimation of sleep and wakefulness in normal subjects and Obstructive Sleep Apnea (OSA) patients. This may provide a useful tool to assess sleep efficiency, maintenance and fragmentation as well as facilitate accurate quantification of obstructive sleep apnea in the home environment (17). Using PAT technology, REM related tonic changes were observed, projecting on attenuated PAT signal amplitude (18). In recent studies, algorithms based on changes in 16 variables of the PAT signal were developed, showing REM detection algorithms in the WatchPAT, a wrist worn device that could be very useful for unattended ambulatory home sleep monitoring. WatchPAT is a self-monitoring device already approved for use by the FDA for the diagnosis of certain sleep disorders (e.g., OSA) through home use in the patient's environment. Patients are asked to place the device on the wrist before going to bed, and place a probe on the finger. During the night they sleep with the device, and in the morning they return it to the box. The innovative method of optimization using a genetic algorithm has been proven to yield robust results in the validation set (19). The Watch-PAT was further validated vs. PSG in a multi-center study that confirmed its detection of sleep stages with moderate agreement to PSG in normal subjects and OSA patients (20).

## Case Presentation

Sixty-three years old woman, suffering from Recurrent Depressive Disorder (F33). Patient is married with three children and does not suffer from psychiatric comorbidity including substance abuse. Physically, recurrent urinary tract infections were recorded on outpatient follow-ups by an urologist, no active infection was present during our research recruitment or follow-up. The patient does not take daily medications, other than the psychopharmacological treatment. The patient experienced her first MDD episode at the age of 39, and suffered from seven recurrent episodes since then. Each episode lasted between 4 weeks and several months, in two of the episodes she was severely depressed and required hospitalization and electroconvulsive (ECT) treatment. Between episodes as well as at the time of recruitment, she was in remission. Her psychopharmacological treatment at time of recruitment included PO TAB Sertraline 100 mg X 2/d, Mirtazapine 30 mg X 1/, Clonazepam 1 mg X 2/d, and P.O TAB Quetiapine XR 100 mg X 1/d. No recent changes in her medications was made.

She was recruited as part of a long-term follow up study, including weekly WatchPAT device recording (which the investigators were blinded from), and a monthly clinical evaluation. WatchPAT is a wrist-worn device, designed for ambulatory Sleep Apnea tests, capable of detecting REM sleep through analysis of Heart Rate and Peripheral Arterial Tone. During our study, the patient placed the device on her wrist before going to bed, as well as placing a probe on her finger. During the night she slept with the device, and in the morning returned it to the box.

Her baseline MADRS score (used as inclusion criteria) was four, compatible with state of depression in remission. She was also evaluated for BDI-II score (used for follow up), with a result of six [cutoff score used to identify potentially clinically significant depression is 0–13 to indicate minimal or no depression; 14–19, mild depression; 20–28, moderate depression; and 29–63, severe depression (21)].

After signing the informed consent forms for participation in the study, she received a WatchPAT device, and began follow up.

**Figure 1:**
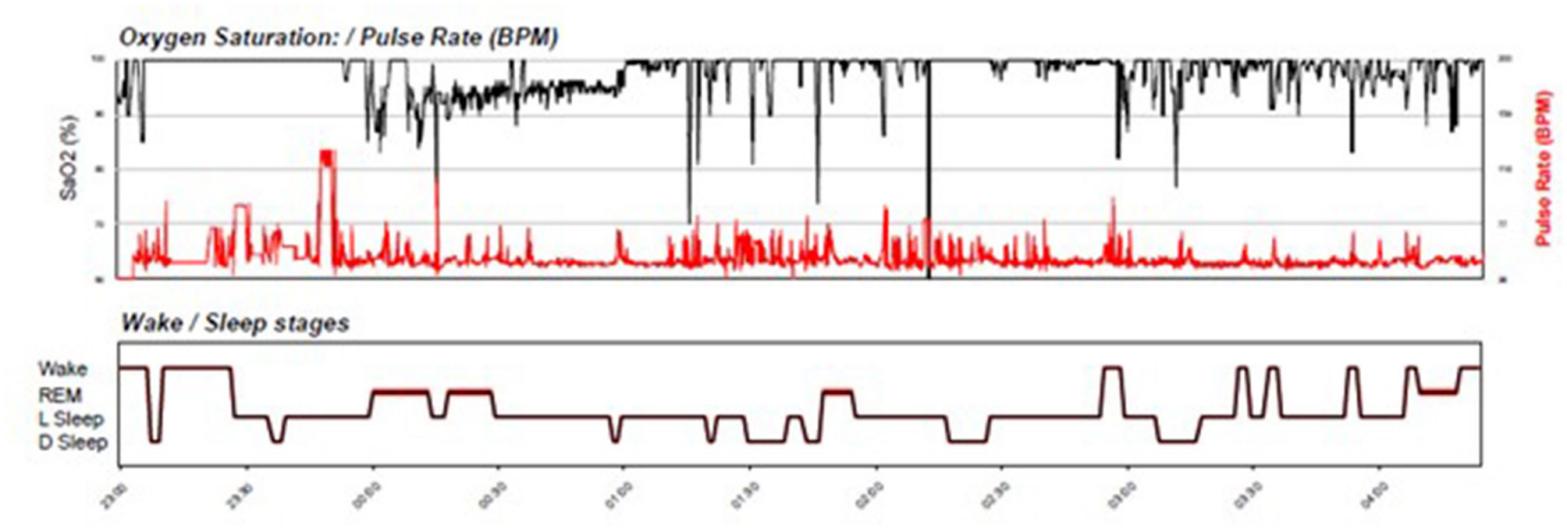
Raw sleep indices measured in sleep on a given night.

**Figure 2:**
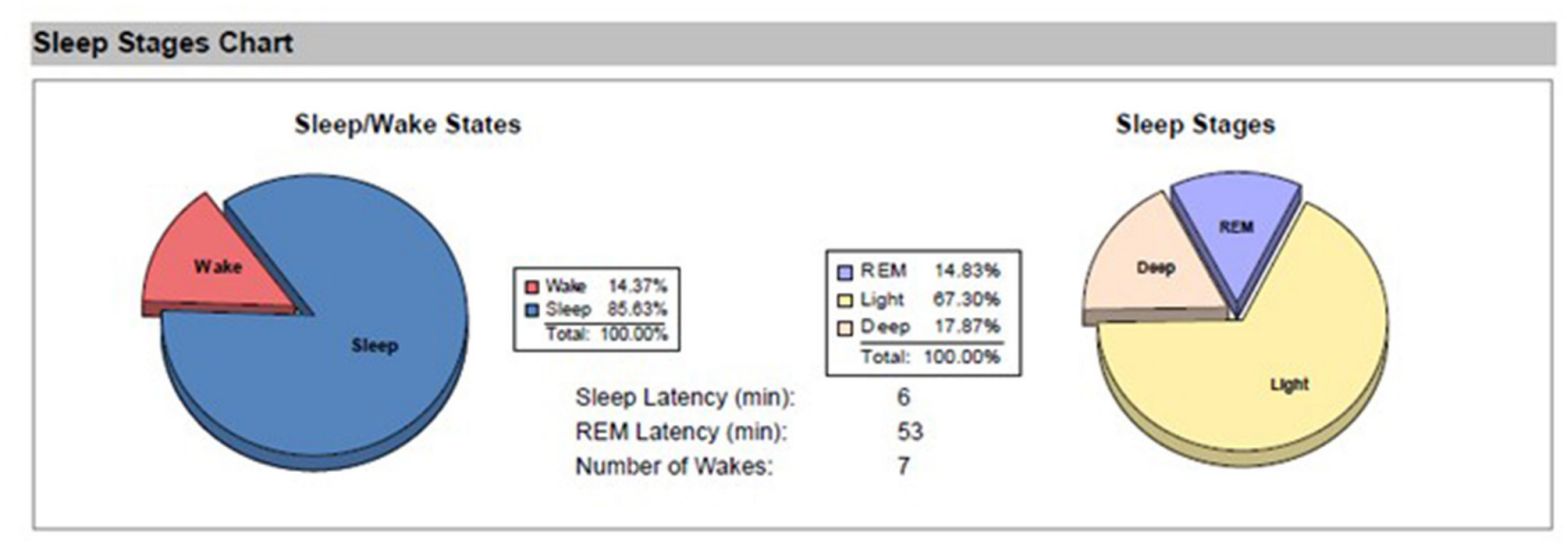
Sleep stages chart on a given night.

Her first sleep study showed the following results, indicating normal sleep variables—REM Latency (min): 117, Sleep Latency (min): 15, Proportion of REM sleep 19.1%, Total Sleep Time 6 h 36 min (examples of data obtained from sleep tests in are presented in Figures 1, 2).

**Figure 3:**
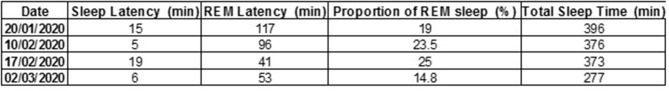
Sleep indices measured during the study.

**Figure 4:**
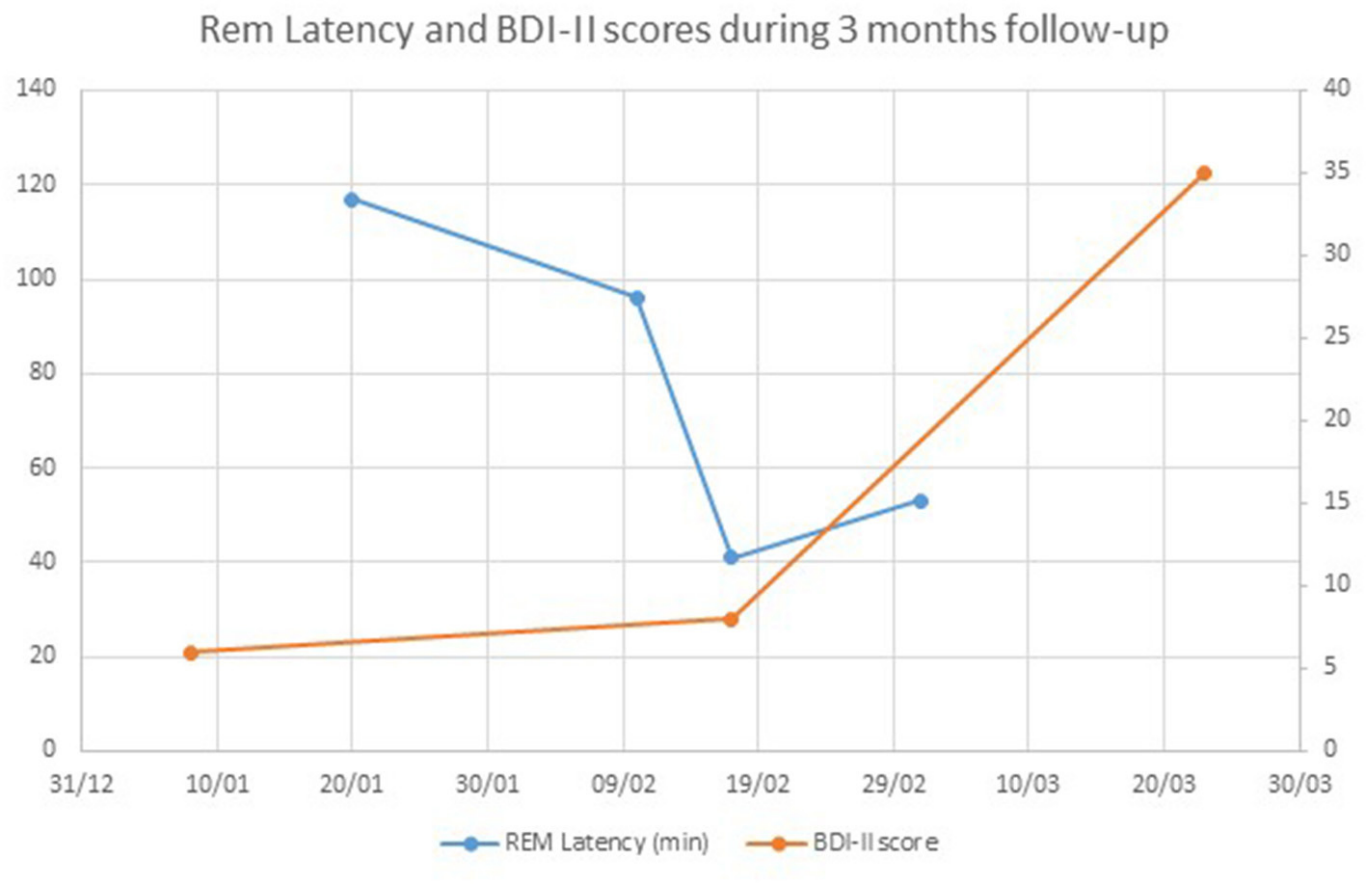
Rem Latency and BDI-II scores during 3 month follow-up.

As shown in Figures 3, 4, Rem Latency was gradually reduced over the following weeks, followed by a significant reduction to 53 min. At the same time, the BDI-II data obtained showed no significant change in self-report. She later complained of lack of energy and asked to stop using the monitoring watch. After a month of subsequent BDI-II follow-up, significant depressive recurrence was discovered and the study was discontinued. Of notice, in several consecutive follow-ups, the patient refused to change her medications, reporting only subjective feeling of anxiety, relating them to the Covid-19 crisis. She agreed to change her psychopharmacological treatment only a month later, and begun reporting subjective improvement. Interestingly during the Covid-19 crisis, she did not leave her house, therefore ambulatory sessions were done using telemedicine platform.

## Discussion and Conclusions

To our knowledge, this is the first study to show long-term follow-up of a high-risk patient for recurrence of depression while measuring objective parameters of sleep. The significant problem of sleep data monitoring applicability is being solved in this preliminary study by using a practical, simple sleep device that could be used in ambulatory setting. This Case study should lead to further investigation whether this sleep monitoring tool could be used as a regular follow up instrument, and further more as a preventive tool for recurrence of major depressive episodes.

As written, the existing data shows strong correlation between major depressive disorders, sleep disturbance, and sleep architecture changes. However, many questions remain unanswered. While few studies managed to show REM sleep alteration in depressed patients, whether these alterations precede symptomatic change remains unknown. Furthermore, there is no sufficient conclusive information regarding sleep changes in patients with recurrent MDD while in remission. Another question that needs to be answered is whether a combination of several sleep changes can predict which patient is in a greater risk for relapse, and what is the time interval between such monitored changes and appearance of subjectively reported depressive symptoms.

The patient presented in this case study has been suffering from recurrent depressive episodes for years. As we have shown, the patient began to show a Rem Latency shortening about a month before reporting a subjective change. Noticeably, even after she reported the change and the research team contacted her, she continued to report the symptoms as part of a general “anxiety” state and was initially unwilling to make any changes to her psychopharmalogical therapy.

This study suggests that some quantifiable biologic changes may precede the patient's basic experience and allow us to provide an initial, better response. As we have shown in the literature review, depressive episodes are best treated as early as possible, and the initial results demonstrated in this study require further research and discussion.

It is important to note that another patient who suffered a depressive episode during the study stopped using the device about a month before relapsing, so it was impossible to observe any changes in sleep architecture. However, it is likely that the changes in patient's compliance in using the WatchPAT device could by itself be regarded as a measure in clinical evaluation.

Another measure that we could be testing in the future using the WatchPAT device is heart rate variability, known to be an interesting biomarker for major depression, possibly creating a combined index. After the clinical study is completed, an attempt will be made to analyze the data in this direction. In the future, we would also like to check the feasibility of using this device for patients with first episode of major depression. This device could be helpful in deciding the optimal time to make treatment changes as well as enable long term monitoring.

In summary, this case report describes ambulatory sleep monitoring, using innovative technology, of a woman at high risk of recurrent depressive episodes. According to the results presented in this case, the course of the disease could seemingly be predictable. So that more intensive treatment and monitoring could be started. This is before the patient's report of subjective mood decline. We hypothesize that the duration of depressive episodes could be shortened or prevented all together if treatment was based on sleep monitoring data. No action items should be drawn from a case report, but it does call for a necessity to complete the extensive research currently underway in our institution, and to further expand research in the field.

## Data Availability Statement

The raw data supporting the conclusions of this article will be made available by the authors, without undue reservation.

## Ethics Statement

The studies involving human participants were reviewed and approved by Clinical Trials Registration—ID Number: MOH_2019-10-28_007458 Registry Name and URL: https://my.health.gov.il/CliniTrials/Pages/MOH_2019-10-28_007458.aspx. The patients/participants provided their written informed consent to participate in this study. Written informed consent was obtained from the individual(s) for the publication of any potentially identifiable images or data included in this article.

## Author Contributions

All authors listed have made a substantial, direct, intellectual contribution to the work and approved it for publication.

## Conflict of Interest

The authors declare that the research was conducted in the absence of any commercial or financial relationships that could be construed as a potential conflict of interest.
